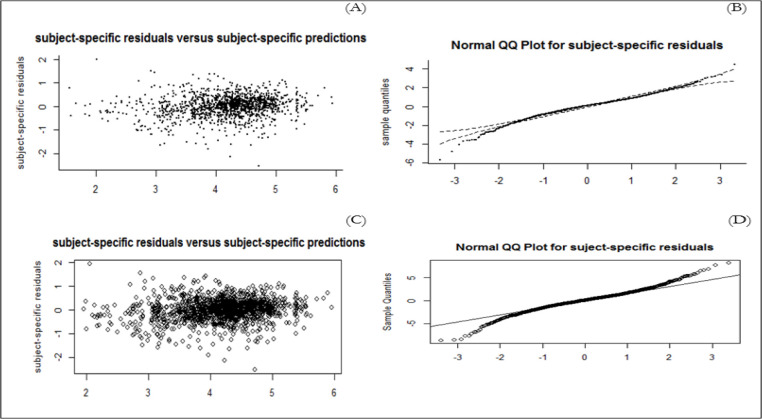# Predicting the Survival of AIDS Patients Using Two Frameworks of Statistical Joint Modeling and Comparing Their Predictive Accuracy

**Published:** 2020-05

**Authors:** Fatemeh KHORASHADIZADEH, Hamed TABESH, Mahboubeh PARSAEIAN, Habibollah ESMAILY, Abbas RAHIMI FOROUSHANI

**Affiliations:** 1. Department of Epidemiology and Biostatistics, School of Public Health, Tehran University of Medical Sciences, Tehran, Iran; 2. Department of Medical Informatics, School of Medicine, Mashhad University of Medical Sciences, Mashhad, Iran; 3. Social Determinants of Health Research Center, Mashhad University of Medical Sciences, Mashhad, Iran

**Keywords:** ROC curve, HIV, Joint latent class model, Shared random effect model

## Abstract

**Background:**

The present study aimed to estimate the survival of HIV-positive patients and compare the accuracy of two commonly used models, Shared Random-Effect Model (SREM) and Joint Latent Class Model (JLCM) for the analysis of time to death among these patients.

**Methods:**

Data on a retrospective survey among HIV-positive patients diagnosed during 1989–2014 who referred to the Behavioral Diseases Consultation Center of Mashhad University of Medical Sciences was used in this study. Participants consisted of HIV-positive high-risk volunteers, referrals of new HIV cases from prisons, blood transfusion organization and hospitals. Subjects were followed from diagnosis until death or the end of study. SREM and JLCM were used to predict the survival of HIV/AIDS patients. In both models age, sex and addiction were included as covariates. To compare the accuracy of these alternative models, dynamic predictions were calculated at specific time points. The receiver operating characteristic (ROC) curve was used to select the more accurate model.

**Results:**

Overall, 213 patients were eligible that met entry conditions for the present analysis. Based on BIC criteria, three heterogeneous sub-populations of patients were identified by JLCM and individuals were categorized in these classes (“High Risk”, “Moderate Risk” and “Low Risk”) according to their health status. JLCM had a better predictive accuracy than SREM. The average area under ROC curve for JLCM and SREM was 0.75 and 0.64 respectively. In both models CD4 count decreased with time. Based on the result of JLCM, men had higher hazard rate than women and the CD4 counts levels of patients decreased with increasing age.

**Conclusion:**

Predicting risk of death (or survival) is vital for patients care in most medical research. In a heterogeneous population, such as HIV-positive patients fitting JLCM can significantly improve the accuracy of the risk prediction. Therefore, this model is preferred for these populations.

## Introduction

The acquired immunodeficiency syndrome (AIDS) continues to be a major global public health issue. Since the start of the epidemic, an estimated 77.3 million people have become infected with HIV and 35.4 million people have died of AIDS-related illnesses. In 2017 an estimated 36.9 million people were living with HIV with a global HIV prevalence of 0.8% among adults. The vast majority of people living with HIV are located in low-and middle- income countries. In Iran, there were 66,000 (37,000–120,000) people living with HIV in 2016 with 5,000 (1,400–13,000) new HIV infections and 4,000 (2,500–6,200) AIDS-related deaths (1).

In many medical and epidemiological researches, patients are often followed up over time and longitudinal measurements are recorded until the time to event of interest (2). In such studies, the association between the survival of patients and longitudinal markers are common of interest. For example, in AIDS clinical trials, CD4 count is the most important clinical measurement that indicates disease progression among HIV/AIDS patients earlier than disease or death (3). We can use this extra information to improve the accuracy of survival prediction (4–6). A common framework to model this type of data is to jointly model the longitudinal trajectory of the marker and the time to event.

There are several advantage of the joint models in the literature. Modeling the longitudinal biomarkers and time-to-event data separately can lead to biased estimates when the longitudinal process is correlated with time-to-event process (7). Joint models can improve the efficiency of statistical inferences, prediction and reduces bias by accounting for the association between the marker and the time-to-event (8,9). Moreover, the one of important advantage of joint modeling is that the impact of each covariate in the longitudinal model and survival model can be examined separately (10). Therefore, joint modeling is a powerful methodology that becoming increasingly essential in cancer, AIDS, and other medical studies not only with regard to better understanding disease processes but also in the growing field of personalized medicine (11–13).

Two commonly used joint models in the literature are Shared Random Effect Model (SREM) and Joint Latent Class Model (JLCM) (14,15). A fundamental assumption of SREM is that the population is homogeneous, i.e. all individuals follow a single mean trajectory. However, in many medical fields, patients consist of some heterogeneous subgroups that rule out this assumption. This heterogeneity may be due to unobserved risk factors such as gene factors or underlying diseases (16–18). To overcome this limitation, JLCM assumes that the population consists of several homogeneous latent subgroups in which the subjects share the same marker trajectory and the same risk of the event. While the use of this model in the cases of heterogeneous population can increase the accuracy of the prediction, few studies have used this model (17,19).

In the present study, we aimed to estimate the survival of HIV-positive patients by joint modeling of time to death and longitudinal CD4 marker. Since progression of many diseases such as HIV/AIDS is heterogeneous among patients yielding different subpopulations, we have used a JLCM in the analysis of this data. Furthermore, we compared the accuracy of this model with SREM in discriminating between patients who will and patients who will not experience the event of interest. To our knowledge, the accuracy comparison of these two models has not been investigated among HIV-positive patients, using history of time-to-death and CD4 measurements, and given that interest is on predicting death within a given time window of interest.

## Materials and Methods

### Study design and participants

This study was a retrospective survey among HIV-positive cases diagnosed during 1989–2014 who referred to the Behavioral Diseases Consultation Center (BDCC) of Mashhad University of Medical Sciences in the Khorasan-Razavi Province, Iran. Participants included in the study consisted of 1) high-risk behavior volunteers with positive HIV/AIDS test; 2) referrals of new HIV cases come from various organizations such as, Mashhad prisons, blood transfusion organization and hospitals. All patients were followed from diagnosis until death, loss to follow-up, or the end of the study (Aug 22, 2014).

For all participants after pretest counseling, a blood specimen was collected. Initially, a rapid test was performed as a screening. If the result of the rapid test was positive, ELISA and Western Blot testing was done as the confirmatory test following the national HIV testing algorithm (20). All subjects completed a structured questionnaire in a face-to-face interview. Patient’s information was strictly confidential. Date of HIV diagnosis was identified as the date when a patient was first diagnosed with HIV. Date and cause of death were extracted from death registration system. The subjects were included to participants of Iranian nationality who had positive confirmatory HIV-test results and recorded at least two CD4 measurements.

The current study was approved by the ethical committee of Mashhad University of Medical Sciences in Iran (IR.MUMS.REG.1392.807).

### Statistical analysis

Time-to-death was computed as the time elapsed between diagnosis and death due to HIV/AIDS in years. Deaths due to other causes were considered as censor. The survival times were right-censored for subjects that were still alive at the end of the study. Because of the shape of distribution of CD4 cell count was right-skewed; therefore we used the $\text{CD}4^{\frac{1}{4}}$ cell count values (6). The subjects with less than two CD4 measurements were removed from the study list wise. We included the same covariates (age, sex, addiction) in both joint models. These covariates had no missing values.

The data was analyzed using joint modeling of longitudinal marker (CD4 cell count) and time-to-event (HIV death). The three steps for defining a joint model were: i) a model for the marker trajectory, usually a mixed model; ii) a model for the time-to-event, usually a proportional hazard model; and iii) linking both models using a shared latent structure (17). The baseline hazards, *λ*_0_(*t*), were parameterized by proportional Weibull hazard functions for both models. Estimation of models’ parameters was based on maximization of the log-likelihood using the robust Marquardt algorithm.

### Shared Random-Effect Model

First, we fitted a linear mixed model for longitudinal sub-model and a proportional hazard model for survival sub-model. We let *Y_i_*(*t_ij_*) denote the longitudinal response for the *i* th patient (*i* = 1,…,*n*) obtained at different time points *t_ij_* > 0,(*j* = 1,…,*n_i_*).

$$\begin{array}{l} Y_{i}(t_{ij})=\beta_{0}+\beta_{1}\mathrm{se}x_{i}+\beta_{2}\mathrm{addictio}n_{i}\\ \;\;\;+\beta_{3}\mathrm{ag}e_{i}+b_{io}+(\beta_{4}+b_{i1})\\ \;\;\;\;\times t_{ij}+\varepsilon_{i}(t_{ij})\\ =m_{i}(t_{ij})+\varepsilon_{i}(t_{ij}) \end{array}$$

Where the *β* parameters are fixed-effects and *b_io_* and *b_i_*_1_ parameters are random-effects having a bivariate normal distribution with mean zero and covariance matrix *B*, i.e., (*b_i_*_0_,*b_i_*_1_)∼*N*(0,*B*). Random-effects were included to incorporate individual variation in the intercept and linear slope. The parameters *β* were called fixed-effects.

The error terms *ε*_i_(t_ij_) were assumed to come from a normal distribution with mean zero and variance *σ*^2^. The random-effects were assumed independent of the error terms. We considered a proportional hazard model for survival analysis. SREM flexibly links the longitudinal and the survival process via the random effects (*b_io_* *and b_i_*_1_) as follows (21–26):

$$\begin{array}{l} \lambda_{i}(t)=\lambda_{0}(t)\exp(\alpha_{1}\mathrm{sex}+\alpha_{2}\mathrm{addictio}n_{i}\\ \;\;\;+\alpha_{3}\mathrm{ag}e_{i}+\alpha_{4}b_{io}+\alpha_{5}b_{i1}) \end{array}$$

### Joint Latent Class Model

Second, we modeled a JLCM to distinguish different profiles of CD4 trajectories among HIV-positive patients. Our JLCM had three ingredients: class membership, the longitudinal biomarker trajectories, and the hazard for the time-to-event process. We assumed that each patient belongs to one of *g* latent classes. Patients with similar characteristics and trend of biomarker were assumed to belong to the same class. After fitting the model, each patient is assigned to the class with higher posterior probability of membership. A shared random effect model is used to describe the individuals’ trajectories within each subpopulation (2). Conditionally on each latent class (*g*), we modeled the CD4 trajectory of subject i by

$$\begin{array}{l} Y_{i}{\left(t_{ij}\right)}_{{|}_{c_{i}=g}}=\beta_{0g}+\beta_{1}\mathrm{se}x_{i}+\beta_{2}\mathrm{addictio}n_{i}\\ \;\;\;+\beta_{3}\mathrm{ag}e_{i}+b_{io|c_{i}=g}+\\ \;\;\;\left(\beta_{1g}+b_{i1|c_{i}=g}\right)\times t_{ij}+\varepsilon_{i}(t_{ij}) \end{array}$$

With $\left(b_{i0|c_{i}=g},b_{i1|c_{i}=g}\right)∼N\left(0,\sigma_{g}^{2}B\right)$

Here, the latent class membership for each subject *i* was defined using a categorical latent variable *c_i_*, which equals g if subject *i* belongs to latent class *g* (*g*= 1, …, *G*). We considered an unstructured variance-covariance matrix of the random effects which are the same over latent classes. Hazard of death was modeled as follows:

$$\begin{array}{l} \lambda_{i}(t|c_{i}=g)=\lambda_{0}(t)\exp(\alpha_{1}{\mathrm{sex}}_{i}\\ \;\;\;\;+\alpha_{2}\mathrm{addictio}n_{i}+\alpha_{3}\mathrm{ag}e_{i}) \end{array}$$

Moreover, we assessed the conditional independence (CI) assumption in JLCM. This fundamental assumption considered independence between the longitudinal measurements and the time-to-event given the latent classes. Next step was to obtain the optimal number of classes that could explain the heterogeneity of the population. We successively estimated models with 1, 2, 3 and 4 latent classes. The optimal number of classes was defined by the model with the lowest BIC (27).

### Model comparison using dynamic prediction accuracy

An important characteristic of joint models approach, which gains increasing interest in recent years, is that predictions have a dynamic nature, that is, as time progresses, additional longitudinal measurements are recorded for the patient, and the predictions can be updated utilizing the new information. Therefore, we can obtain the dynamic personalized prediction of future longitudinal outcome trajectories and risks of survival events at any time, given the subject-specific outcome profiles up to the time of prediction (24,28).

We computed subject-specific predictions at specific times *s* = 1, 1.5, 2, 2.5, 3, 3.5, 4, 4.5, 5, 5.5, 6, 6.5, 7, 7.5 and 8 years with a prediction window of *t* = 3 years for all subjects in dataset. For each time *s*, dynamic predictions were computed for JLCM and SREM. To compare the predictive accuracy of a joint model, we computed ROC curves based on dynamic prediction. Models were fitted using the “lcmm” and “frailtypack” packages in R.3.4.4.

## Results

After excluding non-eligible subjects the final analyses consisted of 213 HIV-positive patients that met the eligibility criteria over the period 1989–2014. The median age of patients was 37.94 yr (interquartile range (IQR) =10.82). The higher percentage of patients were male (81.7%) with a median age of 38.28 yr (IQR=10.27). The median of baseline CD4 cell counts was 397 cells/*mm*^3^ (IQR=380). Follow-up times varied between individuals and in total 1426 measurement occasions were available with a median number of visits per subject of 5 (IQR= 6). During the follow-up, 51 patients (23.9%) died.

Table 1 shows the result of fitting SREM. As expected, the coefficient for the time effect has a negative sign indicating that on average the square root CD4 cell counts declined in time (*β̂*_4_ = −0.079, *P*<0.001). For the random effects, we could observe that there was greater variability between patients in the baseline levels of CD4 (*σ*^2^ = 0.466) than in the evolutions of the marker in time (*σ*^2^ = 0.009). Here two processes (longitudinal and survival) were linked via the random intercept and slope of the longitudinal trajectory. This association was significant for the random intercept implying that with the increase of individual deviation from the population average CD4 counts, the risk of death decreased as well (*α̂*_4_*b*0__ = −1.013, *P*<0.001). Moreover, this association for the random slope was significant (*α̂*_5_*b*1__ = −6.410, *P*<0.001) and established a need for a joint model to analyze the data. Sex and addiction variables were significantly effective on the average CD4 counts but in survival part only age was significant.

**Table 1:** Estimation results of SREM model

***Model***	***Parameter***	***Estimate***	***Standard Error***	**P-*value***
	Longitudinal Part			
	Intercept	4.825	0.338	<0.001
	Time (years)	−0.079	0.010	<0.001
	Sex (female)	0.263	0.096	0.006
	Addiction^*^	−0.197	0.090	0.029
	Age (years)	−0.010	0.009	0.225
	Survival Part			
	Sex (female)	−1.243	0.756	0.100
	Addiction^*^	−0.944	0.771	0.220
	Age (years)	−0.045	0.021	0.032
	(*α̂*_4_*b*0__	−1.013	0.269	<0.001
	*α̂*_5_*b*1__	−6.410	1.228	<0.001

* Addiction: subjects without history of addiction

To assess the optimal number of classes in JLCM, we specified four models with differing numbers of classes. The JLCM with the lowest BIC included two latent classes but the conditional independence assumption was rejected for this model so that the model with three latent classes for which the CI assumption was not rejected (*P*=0.5801) was preferred (Table 2).

**Table 2:** Comparison of BIC of JLCMs, with a total number of classes varying from 1 to 4

***Joint Latent*** ***Model***	***Likelihood***	***P***	***BIC***	***Score Test (P-value )***	***Latent class proportion (%)***
G=1^*^	−1547.86	14	3170.77	22.326(<0.001)	100
G=2	−1519.09	21	3150.76	14.505(<0.001)	(84.51,15.49)
G=3	−1505.32	28	3160.76	1.089(0.5801)	(9.39,37.56,53.05)
G=4	−1489.96	35	3167.57	2.092(0.3513)	(12.68,29.58,2.35,55.4)

* G= number of classes

After identifying the number of latent classes, we estimated the full model which included the covariate variables. As the aim was to propose a dynamic prognostic tool, we chose to include the same covariate variables in all parts of the JLCM and SREM. Table 3 reports the estimates for fitting JLCM. Similar to SREM, the coefficient for the time effect has a negative sign indicating that on average the square root CD4 cell counts declined in time in three classes. This decrease in CD4 cell counts over time in class 1 is worse than other classes (*β̂*_11_ = −0.388) which indicates deterioration in the health of individuals in this class. Men had higher hazard rate than women (exp(2.375)=10.75, p-value=0.010). Also, the CD4 counts levels of patients decreased with increasing age (*β̂*_2_ = −0.016, *P*=0.009).

**Table 3:** Estimation results of JLCM for 3-class model

***Model***	***Parameter***	***Estimate***	***Standard Error***	**P-*value***
Fixed effects in the class-membership model^*^				
	intercept class1	1.211	1.835	0.509
	intercept class2	4.563	1.835	0.013
	Sex class1 (female)	−1.495	1.092	0.171
	Sex class2 (female)	−2.186	1.298	0.092
	Addiction^**^ class1	0.819	1.008	0.416
	Addiction^**^ class2	0.300	1.188	0.801
	Age class1 (years)	−0.070	0.043	0.102
	Age class2 (years)	−0.118	0.043	0.007
Survival Part				
	Sex (female)	−2.375	0.918	0.010
	Addiction^**^	−0.341	0.813	0.675
	Age (years)	0.021	0.029	0.481
Longitudinal Part				
	intercept class1	4.370	0.323	<0.001
	intercept class2	5.263	0.220	<0.001
	intercept class3	5.107	0.297	<0.001
	Time class1(years)	−0.388	0.070	<0.001
	Time class2 (years)	−0.163	0.031	<0.001
	Time class3(years)	−0.016	0.016	0.320
	Sex (female)	0.204	0.151	0.177
	Addiction^**^	−0.176	0.141	0.212
	Age (years)	−0.016	0.006	0.009

* the class of reference is the last class

** Addiction: subjects without history of addiction

**Fig. 1:**
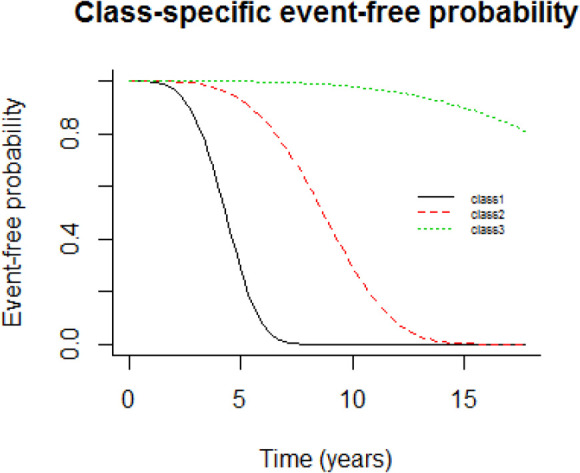
Class-specific predicted survival curves according to time in the 3-class model

Class-specific predicted survival functions, displayed in Fig.1 show a large latent class (class 3) representing 53.05% of the subjects with a very small risk of death over years.

Class 2 and class 1 (representing respectively 37.56%, 9.39% of the subjects) correspond to different profiles of CD4 trajectory associated with risks of death from moderate to intense. Based on our interpretation of the results, we labeled these classes as “High Risk” (class1), “Moderate Risk” (class2) and “Low Risk” (class3). We also examined how precisely the 3-class latent model assigns subjects to classes. The 3-class latent model provided very good discrimination with mean maximal posterior probabilities of subjects classified respectively, 0.84, 0.80 and 0.84 for classes 1 to 3.

**Fig. 2:**
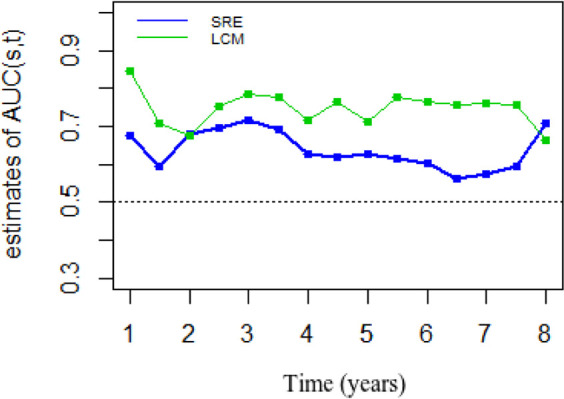
Comparison of predicted accuracy of the two joint models within time window (s,s+t) when s={1,1.5,2,2.5,…,8} and t=3 years

To verify whether the model predicted correctly the number of observed events, we considered the martingale residuals. In a well model, a smoothing curve added to a graph should be approximately overlapping with the horizontal line y=0 (Fig. 1 in supplementary) (29). Moreover, for longitudinal outcome (CD4), marginal and conditional residuals were plotted (Fig. 2 in supplementary). All figures demonstrate a good fit for both the longitudinal and the time-to-event data. We assessed how well the model performs in terms of discriminating between subjects who were going to experience death, and those who were not. We calculated AUCs (s, t) at specific point times and the time windows of interest will be 3 years. Figure 2 shows that estimated AUCs for both models corresponding to the two prediction models were high (range from 0.56 to 0.84). Clearly, dynamic predictions for JLCM had a better predictive accuracy than SREM. The average area under ROC curve for JLCM and SREM was 0.75 and 0.64 respectively.

## Discussion

In present study, we considered two popular approaches of joint modeling of longitudinal data and time-to-event for prediction survival in HIV-positive patients using CD4 cell counts and time-to-death, accounting for individual patient’s heterogeneity. We used dynamic prediction to compare these models and selecting the optimal model. When the history of patient’s information is considered, the accuracy of clinical decisions may be improved. Thus, it is useful to dynamically predict patients’ risk of death using disease history.

So far, most of the literature in the joint modeling have focused on SREM (12,14,24,30). The JLCM and assessment of its power have received less attention. This model considers the population of subjects as heterogeneous that consist of the homogenous classes of subjects that have same longitudinal marker and same risk of event (17,18).

Our finding indicated that predictions of death using repeated measurements of CD4 are better for the JLCM than the SREM. Therefore, JLCM had a good predictive accuracy than SREM and would be more appropriate for this heterogeneous population. The joint model showed that the hazard of death depended on a longitudinal process, i.e., patient’s CD4 count significantly impact on his or her survival time. Moreover, the result of the article confirmed that HIV/AIDS patients’ population was not homogenous. Individuals were categorized in three classes (“High Risk”, “Moderate Risk” and “Low Risk”) according to their health status. This fact enables clinicians to make better medical decisions for the care and treatment of patients in order to increase their survival. Time had a negative effect on CD4 longitudinal measurements in the two approaches. This means that CD4 count decreased with time. The results of this study is consistent with other works on HIV/AIDS dataset (18,31).

Because of the dynamic nature of these models, evaluating the predictive accuracy of joint models using prognostic tools is complex. Recently, there are some studies in this area (6,32, 33). However, few studies have been conducted to compare the accuracy of these two models. Accurate prediction of the future trajectory is helpful for clinicians to monitor patients’ disease progression, make the informative medical decision and can advance the design of future studies (32,34). Moreover, the result of this paper was consistent with a research carried out on prostate cancer. In this work, the accuracy of two models was compared using other predictive accuracy measurements (Brier score (BS) and expected prognostic observed cross-entropy (EPOCE)) (17). We have mainly focused on discrimination (AUC) rather than calibration (BS) because even if a joint model is not well calibrated, there are some approaches used to improve the accuracy of predictions without distorting discrimination (35).

In this study, we only explored the probability of death due to HIV/AIDS. However, it is also possible to predict the competing risk along with the risk of death. The participants of this study were HIV-positive people referring to BDCC for receiving treatment. Due to most patients suffer from addiction or behavioral problems, following-up them and recording CD4 counts has many problems. Therefore, many patients were excluded from the study due to the need for at least two repeated measurement times. Moreover, the results of this study were illustrated using a dataset and may not be generalized to all populations. This was for illustrative purposes only. For future works, we extend this methodology by simulation under various scenarios (different sample size, different missing algorithms).

## Conclusion

Joint modeling of longitudinal biomarkers and time-to-event data for analyzing AIDS clinical trials using CD4 count measurement as an important predictor of survival will result in unbiased and more efficient estimates. Heterogeneity is very common in most societies and in particular in medical research. In practice, patients often have different profiles of the disease. Therefore, ignoring this issue can lead to biased results and misleading. Estimating the prediction of patients based on their demographic, biological, or disease characteristics is an important issue, as it may be used for guiding medical decisions. Therefore, the use of effective and flexible modeling approach, such as JLCM, can help physicians to make better decisions and to obtain a clear picture of diseases for patient-specific treatment strategies and future clinical interventions.

## Ethical considerations

Ethical issues (Including plagiarism, informed consent, misconduct, data fabrication and/or falsification, double publication and/or submission, redundancy, etc.) have been completely observed by the authors.

## Supplementary Figures

Fig. 1:Martingale residual for survival data
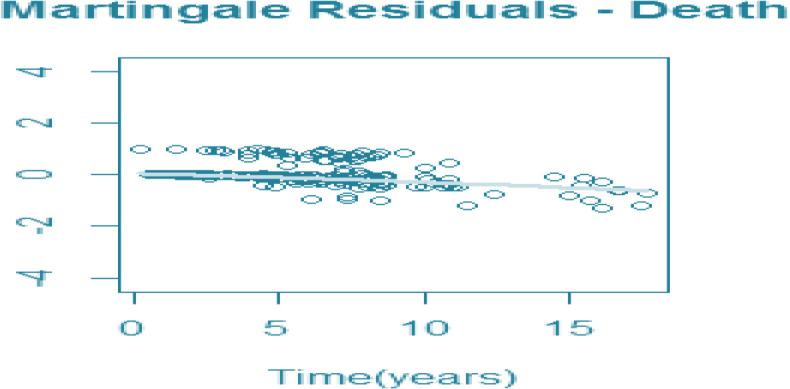

Fig. 2 :(A) subject-specific residuals versus subject-specific predictions from the 3-class JLCM. (B) Normal QQ Plot for subject-specific residuals from the 3-class JLCM (C) subject-specific residuals versus subject-specific predictions from SREM. (D) Normal QQ Plot for subject-specific residuals from SREM